# Dietary Patterns and Nutritional Value in Non-Communicable Diseases

**DOI:** 10.3390/nu16010082

**Published:** 2023-12-26

**Authors:** Charalampia Amerikanou, Chara Tzavara, Andriana C. Kaliora

**Affiliations:** Department of Nutrition and Dietetics, School of Health Science and Education, Harokopio University of Athens, 70 El. Venizelou Ave., 17676 Athens, Greece; amerikanou@windowslive.com (C.A.); htzavara@med.uoa.gr (C.T.)

Non-communicable diseases (NCDs) constitute the leading cause of mortality worldwide, with the four major contributors being cardiovascular diseases (CVDs), cancers, respiratory diseases, and diabetes [1]. Many of the risk factors that contribute to NCDs are modifiable, such as diet, physical activity, smoking, and alcohol use, with diet possessing an essential role in both prevention and management. In recent years, researchers have focused more on the study of dietary patterns instead of single nutrients and foods, suggesting that combined effects can be presented due to synergy or antagonism [2]. Various dietary patterns have been proposed as protective or detrimental for NCDs, with the Mediterranean diet standing out for its association with a lower incidence of NCDs and reduced mortality risk. However, there is still a lot to be explored in the link between dietary patterns and different disease-related factors, making this scientific field very promising.

CVDs are the most common NCDs and the leading cause of death worldwide. The role of diet in CVDs and its risk factors, such as hypertension, diabetes, and dyslipidemia, is pivotal; therefore, the investigation of dietary patterns in CVD and related comorbidities is of high importance. Recently, Dimitriou and colleagues [3] explored the association of dietary patterns, extracted from 1017 Greek individuals, with CVD risk. The Western-type pattern, which included red and processed meat, fried potatoes, and fast foods, was associated with increased CAD risk (OR = 1.20; 95% CI = 1.09–1.32, *p* < 0.001) [3]. In another Greek study, which included 146 Greek metabolically unhealthy obese adults, extracted dietary patterns were associated with several cardiometabolic and lifestyle parameters [4]. More specifically, the Western-type pattern was positively associated with anthropometric indices (fat and waist circumference) and insulin, and the Mediterranean diet-like pattern was positively associated with high-density lipoprotein (HDL) and mental health score, whereas it was negatively associated with depression score [4]. Regarding metabolic syndrome and its association with dietary patterns, a Chinese study with 5426 participants showed that two healthy dietary patterns (“dairy and fruits” and “coarse cereals and soy products”) had protective effects on MetS (OR 0.81 (95% CI: 0.66, 0.98) and 0.74 (95%CI: 0.61, 0.91), respectively) [5].

Apart from dietary patterns, dietary fiber intake and dietary antioxidant capacity in cardiometabolic diseases have recently been investigated. Zhang and his colleagues [6] analyzed data from a longitudinal study (the China Nutrition and Health Database), including 24 h recalls from 2004 to 2015, examining 8307 individuals, and they computed dietary fiber intake. Contrary to other similar studies, whole-grain fiber intake was positively associated with hypertension (hazard ratio (95% confidence interval) (quartile 3 vs. quartile 1) was 1.21 (1.04, 1.40)), but not with other cardiometabolic diseases [6]. A Polish study (n = 5690) evaluated the association between dietary total antioxidant capacity (DTAC), healthy diet quality, and the risk of CVDs [7]. The dietary assessment was based on 24 h dietary recalls, and DTAC was extracted from published databases that use the ferric ion reducing antioxidant potential (FRAP) method to measure the antioxidant potential of foods. DTAC was associated with a reduced CVD odds ratio (OR = 0.593, *p* <0.0001) and a higher Healthy Diet Indicator (HDI). HDI is an index based on the World Health Organization’s recommendations, and it includes the consumption of saturated and polyunsaturated fatty acids, cholesterol, protein, fiber, free sugars, fruits, and vegetables. Also, DTAC was higher in individuals with a higher dietary intake of total polyphenols, antioxidant vitamins, and minerals (*p* < 0.0001) [7].

Another metabolic dysfunction with a high worldwide prevalence is nonalcoholic fatty liver disease (NAFLD). A prospective analysis of 128,695 UK Biobank participants recently revealed a relationship between dietary patterns and the long-term outcomes of NAFLD [8]. Patterns that were prudent and high in whole grains were associated with a lower risk of end-stage liver disease (0.74 and 0.87, respectively) and all-cause mortality (0.86 and 0.94, respectively), whereas a meat-rich dietary pattern had a U-shaped association with all-cause mortality [8]. In a cross-sectional study of 320 Lebanese NAFLD patients, high adherence to a traditional diet was associated with a lower risk of fibrosis [0.18 (0.04–0.85), *p* = 0.031]. The traditional pattern included vegetables, legumes, vegetable oils, nuts, fish, red wine, and cooked rice [9].

Although many studies have addressed the association between diet and cancer risk in recent years, there is a lack of studies on the influence of dietary patterns on cancer therapy response, as well as the combined effect of dietary and genetic factors on cancer risk. Tiberio and his colleagues [10] analyzed the eating patterns of 82 breast cancer patients undergoing chemotherapy and how these can affect positron emission tomography/computed tomography (PET/CT) outcomes related to therapy response. The colon uptake value was correlated positively with drinks (alcohol and spirits; r = 0.33, *p* < 0.01) and foods (red and cured meats; r = 0.25, *p* = 0.04) related to inflammation, and rectum uptake was correlated negatively with anti-inflammatory foods (fruits and vegetables; r = −0.23, *p* = 0.04) [10]. Liu and his colleagues [11] created a polygenic risk score using UK Biobank genetic and dietary data (n = 415,589). A healthy dietary score based on the consumption of fruits, vegetables, grains, fish, and meat reduced the risk of upper gastrointestinal cancer (UGI) [hazard ratio: 0.76 (0.62–0.93), *p* = 0.009]. When a healthy pattern was combined with a low genetic risk, it was associated with a lower risk of UGI cancer compared to a high genetic risk and an unhealthy dietary pattern [11].

The exploitation of dietary patterns in low- and middle-income countries has uncovered a transition of traditional diets towards westernized diets, as well as the contribution of this dietary shift to obesity, malnutrition, and other NCDs. A data analysis of 8957 adults from the 8th Philippine National Nutrition Survey (PNNS) estimated a 30% prevalence of the double burden of malnutrition (DBM), indicating the coexistence of undernutrition and overnutrition in the same population [12]. Seven dietary patterns were extracted in this study, with a rice pattern and a meat and sugar pattern in males and a protein-rich food, cereal, and sugar pattern in females being associated with lower odds of DBM. Vegetable and corn patterns showed an increased risk of DBM in women [12]. In a study aiming to explore the dietary patterns of children in South Africa, aged 1–10 years, and their relationship with socio-demographic factors, a greater adherence to unhealthy patterns in a higher socio-economic status and in the presence of an obese mother was observed [13]. The improvement of children’s eating habits is pivotal not only in low- and middle-income countries but also in children from households with low incomes in developed countries [13]. In the USA, improving children’s dietary intake with healthier foods and beverages has been implemented through the USDA Child and Adult Care Food Program (CACFP) [14]. A longitudinal study of childcare centers participating in CACFP evaluated their meal patterns and concluded that, although CACFP centers followed better nutrition standards than non-CACFP centers, their menu quality had not improved since 2017, when an increase in whole grains, fruits, and vegetables had been proposed [14]. The above indicates that there is a lack of effective strategies towards the improvement of children’s diet quality and eating habits in both developing and developed countries.

The importance of diet is also evident in other common NCDs. Functional dyspepsia affects 10–30% of adults, with food being one of its main triggering factors. A review tried to shed light on whether specific food categories, dietary patterns, or eating habits influence the symptomatology of functional dyspepsia [15]. The authors concluded that, although several foods and patterns have been proposed as dyspepsia triggers, evidence on the association between diet and symptomatology is scarce and inconsistent, highlighting the need for more research in this field [15]. Fibromyalgia is a chronic syndrome with a prevalence of 2–3% in the general population. A systematic review that addressed the relationship between diet and pain in fibromyalgia included 12 studies (11 interventions and 1 observational) and 546 participants [16]. Interestingly, all plant-based and anti-inflammatory diets improved pain measurements, whereas not all gluten-free or elimination/restrictive diets showed statistical significance in pain improvement [16]. The significance of plant-based diets also emerged in a study of cognitive impairment in Chinese adults [17]. In this longitudinal prospective study, 1077 out of 4792 participants developed cognitive impairment, which showed a reverse J-shaped association with BMI, meaning that overweight and obese participants had a decreased risk, whereas underweight participants had an increased risk of cognitive impairment. Interestingly, overweight participants with a higher plant-based diet index had a lower hazard ratio for cognitive impairment than those with a lower index (HR = 0.74; 95% CI 0.57–0.95 vs. HR = 0.87; 95% CI 0.67–1.12). The above suggests that high adherence to plant-based diets enhances the protective effect of overweight on cognitive impairment [17].

Conclusively there is a growing interest in the relationship between diet and dietary patterns with NCD-related parameters. Our Special Issue offers some new data in this field. More research is needed to elucidate the exact mechanisms under which dietary patterns, as well as individual foods and nutrients, exert their effects on NCDs and related clinical, genetic, environmental, and behavioral factors.
